# Traditional Knowledge Evolution over Half of a Century: Local Herbal Resources and Their Changes in the Upper Susa Valley of Northwest Italy

**DOI:** 10.3390/plants13010043

**Published:** 2023-12-22

**Authors:** Naji Sulaiman, Dauro M. Zocchi, Maria Teresa Borrello, Giulia Mattalia, Luca Antoniazzi, S. Elisabeth Berlinghof, Amber Bewick, Ivo Häfliger, Mia Schembs, Luisa Torri, Andrea Pieroni

**Affiliations:** 1 University of Gastronomic Sciences, Piazza Vittorio Emanuele II 9, 12042 Pollenzo, Italy; 2 Faculty of Health Sciences and Wellbeing, School of Pharmacy, University of Sunderland, Sunderland SR1 3SD, UK; 3 Institut de Ciència i Tecnología Ambientals (ICTA-UAB), Universitat Autònoma de Barcelona, 08193 Barcelona, Spain; 4 New York Botanical Garden, New York, NY 14058, USA; 5 Leibniz-Zentrum für Agrarlandschaftsforschung (ZALF) e.V., Eberswalder 84, 15374 Müncheberg, Germany; 6 Department of Medical Analysis, Tishk International University, Erbil 44001, Iraq

**Keywords:** Alps, ethnobotany, food-medicine, infusion, liqueur, rural development, secondary products, tea

## Abstract

Susa Valley, located in the Italian Western Alps, has served as a meeting point for cultural, spiritual, and commercial exchange for a long period of history. The valley’s role as one of the main connecting routes between south and southwestern Europe resulted in its acquisition of a rich traditional ecological knowledge. However, like other Italian mountainous valleys, this valley has suffered from abandonment and depopulation in the past 50 years. Our study aims to investigate the current ethnobotanical medicinal knowledge in the valley and to compare our findings with a study conducted over 50 years ago in the same area. In 2018, we conducted 30 in-depth semi-structured interviews on medicinal plants and food-medicines used in the Susa Valley. We documented 36 species, of which 21 species were used for medical purposes and 15 species were used as food-medicine. The comparison with the previous study on medicinal herbs conducted in 1970 in the valley demonstrated a significant decrease in both the knowledge and use of medicinal plants, which could be attributed to socioeconomic, cultural, and possibly environmental changes that occurred in the past half-century. Our study highlights several promising species for future use as nutraceuticals, food, and medicinal products, such as *Taraxacum officinale*, *Urtica dioica*, and *Artemisia genipi*.

## 1. Introduction

Traditional ecological knowledge (TEK) is vital for local communities and their well-being. TEK has been a focal point of research within the ethnobiological field, and recent developments have witnessed a shift towards a diachronic approach that emphasizes the historical evolution of these corpora of knowledge [1,2,3,4,5,6,7]. The TEK of mountain communities has received more attention in the past decades because of the crucial role of mountains in providing multiple services (i.e., water, pastures/dairy products, timber and non-timber forest products, and leisure) to both highland and lower-land dwellers and urban visitors [8]. Despite their essential contributions, mountain areas are particularly vulnerable to social and environmental phenomena such as depopulation and climate change [9,10]. Therefore, mountain areas have become a focus of important socio-ecological research and public debate [11,12,13]. In recent years, only a few ethnobotanical studies in the Western Alps have focused on the interactions between plant resources and human societies [2,3,4,5,14,15]. These studies have partially discussed how traditional knowledge of plants evolves across cultures over time and space. Understanding the interactions between nature and culture and their transmission mechanisms across generations is essential for the development of strategies to maintain biocultural diversity in the Western Alps [1]. Over the last few decades, the current state of traditional plant knowledge has been undergoing a transition; this refers to the fact that there is continuous erosion and loss. Aswani et al. [16] suggest that the depletion of TEK can be attributable to geopolitical changes such as globalization, modernization, and market integration. Moreover, the modern cultural industries, including advertising and widespread media content and mass communication, have had a massive impact on traditional and folk cultures.

A few studies have addressed TEK related to food and medicinal plants in the Western Italian Alps [1,2,4,5]. In this context, Fontefrancesco and Pieroni [4] investigated the transformation of local botanical knowledge in the upper Sangone Valley of Piedmont. They compared historical and contemporary datasets, revealing qualitative shifts in plant use. Some medicinal plants from natural environments disappeared from use in local practices. At the same time, those from anthropogenic settings were introduced, likely influenced by urban residents who arrived in the late 1970s as original inhabitants left for urban areas. This study highlights the importance of considering the adaptive capacity of TEK systems and how societal and environmental changes impact traditional knowledge in mountain communities. Similarly, Fontefrancesco et al. [5] addressed rural depopulation in the upper Borbera Valley of the Piedmontese Apennines. By comparing historical and contemporary ethnobotanical data, they determined that the use of wild medicinal plants from natural environments had diminished. At the same time, species from anthropogenic areas, or those promoted by the nearby city of Genoa, were introduced or revitalized. These changes were closely tied to shifts in landscape ecology and the local agro-silvo-pastoral system. This research highlights the challenge of preserving TEK when local actors’ expertise is limited to their villages and underscores the need for measures supporting mountain communities in a changing socio-economic landscape.

Focusing on the historical and ethnobotanical literature related to the Western Italian Alps, an interesting study was identified, which was conducted in 1970 and published in the journal *Allionia: Bollettino dell’Istituto ed Orto Botanico dell’Università di Torino* [17]. The study, which is based on the data collected during fieldwork carried out in 30 villages in the Susa Valley (high, middle, and low), was aimed at gathering information directly from the local people about the uses of medicinal herbs and their perceived properties. The work indicated the exact locations where the information was collected for each individual description of a plant and, when available, the scientific and folk names of the plants were recorded. We, therefore, used this study to carry out a diachronic analysis to investigate the current ethnobotanical medicinal knowledge in the Susa Valley and assess the evolution of this knowledge over half of a century, using as a proxy a comparison between the medicinal plants gathered and used by the local community in the 1970s and those gathered and used in 2018.

Our study aims to document the current ethnobotanical knowledge (specifically focusing on both medicinal and food-medicine plant uses) of the Susa Valley, in the NW Italian Alps. We also aim to compare the recorded data with that recorded in 1970. The study attempts to highlight and interpret possible differences in ecological, socioeconomic, and cultural changes/transformations of TEK.

## 2. Materials and Methods

### 2.1. Study Area

**Figure 1:**
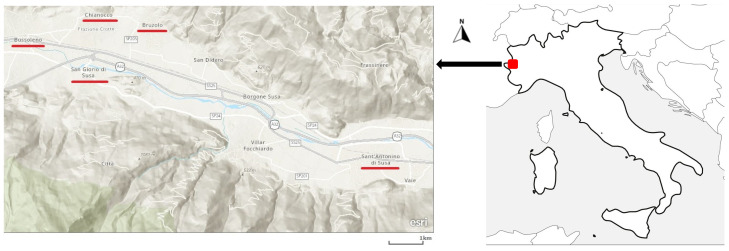
Map of the study area within the map of Italy. Red underlining of villages refers to the locations of the study participants.

The study was conducted in the upper Susa Valley (*Val di Susa*) in the Piedmont region of NW Italy (Figure 1). This Alpine valley serves as a major corridor between Italy and France which has allowed an exchange of goods and cultures since antiquity, and consequently, trade with southwestern Europe. The Susa Valley has been a meeting point for many cultures over the centuries, resulting in an area rich in culture, trade, and tourism. Several languages (Piedmontese, Occitan, French, and Italian) have been co-existing in the valley for a long period of history. The valley has also been a historical transit route for Christians, Muslims, and Jews on their way to the “Holy Land”. Until 2009, the Susa Valley was divided into “*Alta Valle*” and “*Bassa Valle*”. The higher valley, “*Alta Valle*”, was part of France until 1713. The lower valley, “*Bassa Valle*”, was under the influence of the Savoy family [18]. In 2009 the communities of the lower and higher valleys were united. There are more than 90,000 inhabitants in the valley.

Economically, local people have traditionally lived self-sufficiently. Until the 19th century, the economy of the valley was based on agriculture, especially pasturing and related activities like cheese making, milling, and blacksmithing. Aside from those activities, chestnut cultivation was one of the main agricultural activities in the area, producing sweet chestnuts for consumption and environmental value for the landscape. Wood handcrafting was another important economic activity, one which is still practiced in the higher valley. Typical products included, and still are, wine (in the lower valley), cheese, cured meats, chestnuts, potatoes, and fruits [19]. Following the growth of industries in the 19th and 20th centuries, the economic basis of the mountain community of the Susa Valley and many other Alpine valleys has changed existentially with major migrations to the lowlands. This turnover, from a society sustaining itself with its own agricultural products to a modern society based on industry, had great effects on lifestyle, local customs, traditions, culture and the environment. In the 19th century, the textile industry began growing in the valley, and in the 20th century, heavy industry became an important sector. In addition, skiing became increasingly popular in Italy, and winter sports tourism in the Upper Susa Valley became an important economic sector; the Upper Valley even hosted some competitions of the Olympic Winter Games in 2006. This significant socioeconomic, cultural, and landscape change was unwelcome for many local people, as many of them resisted, and still resist, the construction of a high-speed railway that would connect the Italian city of Turin with the French Lyon through the valley [20].

### 2.2. Fieldwork, Data Collection, and Data Analysis

**Figure 2:**
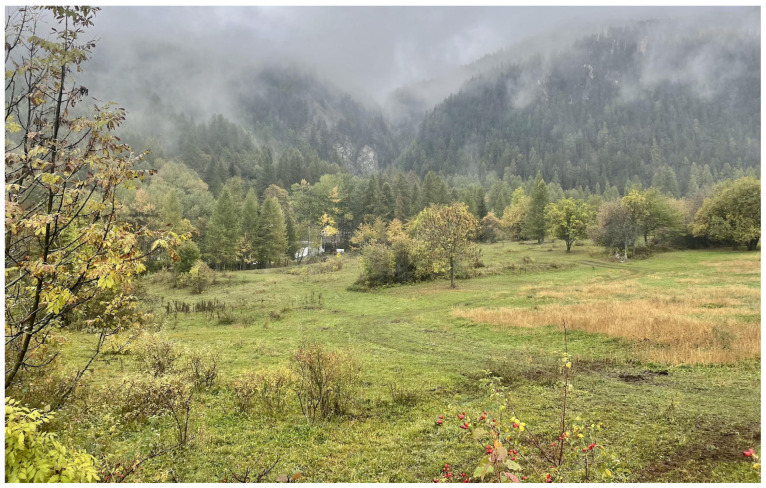
Autumn landscape in the Upper Susa Valley (photo: N. Sulaiman).

The fieldwork was conducted in 2018, mainly in five villages in the Susa Valley (Figure 1). The villages are geographically distributed at elevations between 700 and 1100 m above sea level (Figure 2). The participants were chosen using a combination of random and snowball technique methods in order to attain the advantages of both a general representation of the local communities as well as a targeted search for the knowledgeable respondents [21]. We approached local people in the villages’ streets and local markets (*mercato della terra*) and asked them about the use of medicinal plants in the area. Afterwards, we asked those randomly chosen informants to recommend other potential respondents who may be knowledgeable about medicinal plants. Semi-structured in-depth interviews were conducted with 30 participants (18 men, and 12 women). The ages of our study participants ranged between 24 and 90; however, the majority of them were elderly people, older than 60 years old. Interviewees were first asked to report socioeconomic data related to their residency village, age, and occupation. Afterwards, respondents were asked to list all medicinal plants they used, the vernacular names of the plants, the parts used, the modes of preparation and consumption, the purposes of use, and the additional uses of the plant. We also recorded our observations about the social, cultural, and economic aspects of the area, which were later employed in our qualitative analysis. Verbal consent was always obtained from the study participants, following the Code of Ethics of the Society of Ethnobiology [22]. The interviews were mainly conducted in the Italian language. Plant specimens had been collected, identified, and deposited in a recognized herbarium during previous ethnobotanical fieldwork conducted by some of the authors in the same portion of the Western Alps [4,5]. The botanical nomenclature followed *World Flora Online* [23]. As the main focus of the study is to assess the change in knowledge and use of herbal resources over half of a century, we did not examine the possible differences within our study sample. Instead, we compared our results with the results of a study conducted in 1970 in the same area [17]. However, in order to minimize the limitations related to differences in the applied methodologies between our study and the study from 1970 (e.g., differences in the reported species and the availability of specific species), we conducted a comparison based on the genera level rather than the species level. Additionally, the recorded plant reports were also qualitatively compared with the most comprehensive worldwide wild food-plant compendia [5,7,24].

## 3. Results and Discussion

### 3.1. Diversity of Medicinal Plants Used in Susa Valley

The present study documented 36 plant species, among which 21 species were used for medical purposes, while 15 species were used for food-medicine (Table 1). The reported species belong to 33 genera and 19 botanical families. The most represented families are Asteraceae (ten species), Lamiaceae, and Rosaceae (three species each). These highly represented families include mainly herbs (Asteraceae and Lamiaceae), while the family Rosaceae includes mainly shrubs and trees. Aromatic herbs mainly distinguish the family Lamiaceae, while Asteraceae is characterized by a bitter taste and is well-known for including plants with medical and nutritional properties [25] (and references therein). The most-reported species were *Artemisia genipi*, *Hypericum perforatum*, *Taraxacum officinale*, *Arnica montana*, and *Urtica dioica*, which were reported by more than one-third of our study’s respondents. The study of Lomagno and Lomagno Caramiello [17] reported 99 species (including three mushrooms which are not considered by our analysis). The 96 plant species belong to 76 genera (Table 1).

**Figure 3:**
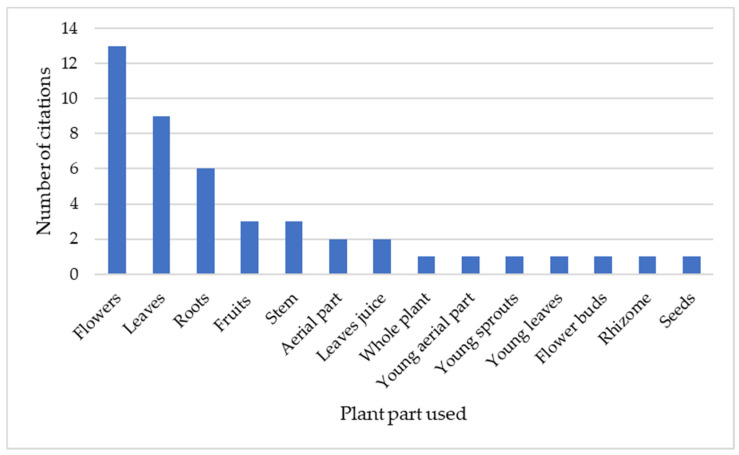
Plant parts used in the reported medicinal plants in the present study in Susa Valley.

We documented in our fieldwork 14 types of plant parts to be used for medical purposes (Figure 3). Flowers were the most reported plant part, with 13 citations by the study’s respondents, followed by leaves (nine reports) and roots (six reports). On the other hand, the study of Lomagno and Lomagno Caramiello [17] documented that “the whole plant” was mostly used among the reported species. We also observed a higher diversity of parts used in 1970 compared to the current uses, as some plant parts reported by Lomagno and Lomagno Caramiello [17] (e.g., resin, petals, bark) are not reported in our present study.

The majority of our reported species are used solely for medical purposes (58%). However, we found that a significant number of the reported species (42%) are used as food-medicine. Some of the most-reported species were consumed as food while their medicinal benefits were targeted. *Urtica dioica* (11 reports), for instance, is frequently used to defeat the deficiency of iron, and for its richness in vitamins E and C. Similarly, *Taraxacum officinale* (12 reports) is consumed as salads or cooked for its benefits to blood and the circulatory system.

Using food plants as medicine and medicinal plants as food has often been reported in the Mediterranean; for instance, Pieroni [24] highlighted that 60% of the medical plants in the upper Garfagnana in Italy also play a role as a food medicine.

Infusion was the main preparation method reported by our respondents; 50% of the reported medicinal plants were prepared as such. The infusion was mostly performed in water (tea), while infusion in liquor, wine, or oil was used with some plants. Similarly, Lomagno and Lomagno Caramiello [17] highlight that tea is the main preparation mode for the vast majority of the species reported in their study. Another main mode of preparation in our present study was consumption as food (cooked, in salads, or eaten raw).

### 3.2. Main Ailment Categories Treated by Medicinal Plants in the Susa Valley

Our present study recorded 41 medical-use reports, corresponding to 41 diseases/health complaints or disturbances treated with medicinal plants in the study area. These diseases were categorized into ten ailment categories based on the classifications of Cook [26]. Specifically, we characterized digestive, respiratory, circulatory, genitourinary, nutritional, musculoskeletal, and skin disorders, as well as disorders of the nervous system, inflammation, and pain (Table 2). The most-reported ailment categories (digestive, respiratory, circulatory, and blood systems) represented more than half of the total number of reports. On the other hand, we see a higher diversity of reports highlighted by Lomagno and Lomagno Caramiello [17], in which five ailment categories had been treated by at least ten plant species for each. Respiratory and digestive system disorders were the most cited category treated by local people half a century ago, which is roughly similar to our current findings in the present study. However, we observe two categories, genitourinary system disorders and musculoskeletal system disorders, that are highly reported in Lomagno and Lomagno Caramiello [17], while they are marginally highlighted by our current study. This could be attributed to the complicated nature of the infections associated with these two categories, which could be better treated by the current health system that has significantly developed over the past half-century.

Several herbal species are employed to treat digestive system disorders; the most cited among them is *Artemisia genipi*. In the Susa Valley and other Alpine valleys, *A. genipi* is used in liqueur preparations [27] made by macerating the plant’s aerial parts (flowers, leaves, and stems) in alcohol for approximately 20–30 days. Next, sugary syrup is added to the alcoholic macerate. The resulting drink, referred to as “génépi” or “génépy”, is used as a tonic and eupeptic beverage, having a bitter taste and unique flavor. Its peculiar sensory properties have been associated with the volatile constituents of the plant and attributed to the sesquiterpene lactone fraction, which conveys stimulating and mood-elevating properties [28]. Over 100 essential oils have been identified in *A. genipi* and other related species (such as *A. umbelliformis* Lam.), mainly produced in leaves and flowers. These are credited with spasmolytic, antiseptic, and sedative properties, and the most abundant of them are used to identify different chemotypes within a species or a group of species [28]. In *A. genipi*, four different chemotypes with distinct olfactory properties have been identified: (a) thujone, the most abundant monoterpene associated with the neurotoxic effect of absinthium; (b) borneol, which gives to the liqueur a woody, camphoraceous note with insect-repellent properties; (c) cineol, which promote a spicy note of cinnamon; and (d) α-,β-Pinenes, which confer a pine resin note [29,30].

The oil infused with the flower-heads of *Arnica montana* is used externally to treat various symptoms related to pain, inflammation, and musculoskeletal system disorders. The plant belongs to a relatively large group of the Asteraceae family, and is rich in sesquiterpene lactones such as helenalin, which have pharmacological and toxicological effects [31]. Other natural products typically found in the flower-head are flavonoids such as quercetin and kaempferol, coumarins, and an essential oil [31,32]. A preparation with *A. montana* showed anti-inflammatory effects in vivo for arthritis [33]. In particular, helenalin seems to interact with many cysteine residues in a number of target proteins, modifying their activity, such as with NFkB and NFAT, two transcription factors orchestrating the inflammation pathways. In addition, its cytotoxic effects have been well documented in several studies, and work analyzing helenalin anticancer potential is still ongoing [34].

*Thymus serpillum* is another plant highly reported in our survey for the treatment of respiratory disease. The oil found in several thyme-related species is carminative, antiseptic, antitussive, expectorant, and spasmolytic. In herbal medicine, it is employed in respiratory disorders such as coughs, bronchitis, and sinusitis, among others [35]. The active principals thymol (a volatile oil) and carvacrol are responsible for the expectorant activity, whereas the flavonoids such as apigenin and luteolin are expected to contribute to the anti-inflammatory and antimicrobial effects of the plant extract [36,37]. The plant extract has also shown in vivo antispasmodic effects [38], further validating its use in respiratory disorders. Interestingly, the essential oil is currently being investigated as a potential food preservative [39].

The boiled leaves, or the decoction, of *Urtica dioca* are used in cases of nutritional deficiency (iron deficiency), and as a source of micronutrients (Vitamin C). The constituents of *U. dioca* are well documented, although it is still unclear which are the components responsible for the pharmacological activities. The root has several lignans, including pinoresinol, secoisolariciresinol, and neo-olivil, which may play a dominant role in inhibiting the interaction between sex hormone binding globulin and 5α-testosterone, a pathway that plays an important role in the development of benign prostate hyperplasia (BPH). The research around *U. dioica* has been focused on BPH symptom relief, and the use for this purpose has been supported by small-scale clinical trials. *U. dioica* extracts are also evaluated as anti-inflammatory agents in arthritis and rheumatism [40,41].

*Taraxacum officinale* leaves are used as a food with medicinal properties. The leaves are eaten raw in salad or cooked like spinach (sauteed in olive oil with garlic), and the flower buds are foraged and preserved in brine and used like capers. The plant is thought to be useful in blood disorders and is a systemically refreshing herb. Analysis of the nutrient content has shown a balanced combination of trace elements, making the plant an interesting source of oligo-elements (calcium, potassium), micronutrients (one of the highest sources in nature of β-carotene), and an array of phytochemicals with potentially beneficial effects [42]. It contains several sesquiterpene lactones with anti-inflammatory and anticancer properties, phenylpropanoids with anti-inflammatory effects, and terpenoid polysaccharides with immune regulation, platelet anti-aggregation activity, and hepatoprotection [43,44,45,46]. It is also an important source of inulin, which has immunostimulatory functions [42]. A growing body of evidence points out that *T. officinale* components such as chlorogenic acid, chicory acid, and taraxasterol have strong hypoglycemic effects, either alone or in combination with other herbs [47,48,49].

Therefore, the phytochemical and pharmacological analyses of plants used medicinally in the Susa Valley has largely provided evidence for their traditional uses and could be a promising economic resource for rural communities.

### 3.3. Changes in the Knowledge and Use of Medicinal Plants over a Half-Century in the Susa Valley

**Figure 4:**
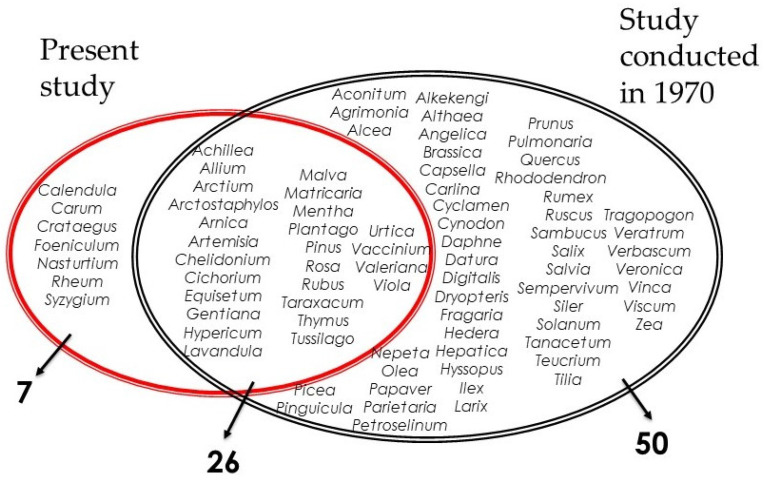
Genera overlapping within our present study and a previous study conducted in the region in 1970.

Compared to the previous study conducted in the region in 1970, the current survey study highlights significant changes in folk-medicine knowledge and associated uses. While our study reports 36 plant species, corresponding to 33 genera, Lomagno and Lomagno Caramiello [17] reported 96 plant species, corresponding to 76 genera. We found that both studies overlap in 26 plant genera which refer to plant genera that are still used in the region (Figure 4). These overlapping genera include the five most cited plant species in our present study (*Artemisia genipi*, *Hypericum perforatum*, *Taraxacum officinale*, *Arnica montana*, and *Urtica dioica*), which were also highlighted for its importance by Lomagno and Lomagno Caramiello [17]. This reflects the cultural importance of these genera in the local ethnobotanical heritage, as a half-century of critical changes could not impact the use and knowledge of these plants.

On the other hand, the use of a few plant genera (seven genera) newly arises in the new survey; this could be attributed to the newcomers to the region who brought with them the use of a few species (e.g., *C. Carvi*, *S. aromaticum*), or due to the recent availability of these species in supermarkets that have spread into the region in the past few decades. This phenomenon was reported in a previous study in Val Borbera (in the same region, Piedmont) on the possible sociodemographic drivers behind the differences in plants, parts used, and modes of preparation in the region. Fontefrancesco et al. [5] observed that the aerial parts of ramson (*A. ursinum*) were never gathered or used in the past in the study area, but city visitors from Genoa introduced a few years ago the custom of collecting and using this plant as a substitute for garlic (and basil leaves) in one of the most iconic Ligurian culinary preparations: pesto. The introduction “from the city” of the use of *A. ursinum*, which grows widely in beech woodlands in spring in the upper Borbera Valley, testifies to a phenomenon that is well known in southern Europe, where this plant was introduced a few decades ago as a panacea, and also possibly thanks to the popularity it achieved when the books by Austrian herbalist Maria Treben (1907–1991), especially *Health Through God’s Pharmacy,* which strongly advocated its use, were translated and became best-sellers in many countries. Similarly, we observed a similar dynamic for some species (e.g., *A. ursinum* and *Lepidium sativum*) that were not documented in the study conducted by Lomagno and Lomagno Caramiello [17]. We can assume that their collection and consumption have probably started in recent decades, following the socioeconomic and demographic changes that have occurred in the Susa Valley.

Figure 4 also illustrates the crucial decrease in the number of medicinal plants used in the region, where the use and knowledge of 50 genera, which correspond to 73 plant species, have been abandoned. This large number of plant species reported solely in 1970 could be attributed to, in addition to the erosion of traditional knowledge, the differences between the sampled participants in the two studies. While we interviewed normal local people, Lomagno and Lomagno Caramiello [17] mainly interviewed herbalists; this can be observed by the inclusion of some described species that are typically used in scholarly medicine, such as *Aconitum* spp., *Veratrum* spp., and *Dryopteris* spp., given that some of them have a level of toxicity and need to be used carefully. The decrease in traditional knowledge during a half-century’s frame may be a reflection of the vast socioeconomic, cultural, urban, and environmental changes that have occurred in the region. Knowledge about herbs and plants of the valley once was necessary, as people in the valley were self-sufficient. People foraged plants and used them for cooking and as medicine to cure themselves. With the industrial growth in the Piedmont Plain and Turin area, which has attracted new human resources for labor from the mountain valleys, the decrease in agricultural activity, and, most notably, the decrease or even loss of dependence on this knowledge of local plants to sustain local populations, the number of practices linked to local plants and the number of people making use of them have shrunk. Food and medicinal products have been institutionalized, and non-prescribed drugs have been advertised on a massive scale. This is in addition to the spread of supermarkets and pharmacies that provide all necessities, independent of ethnobotanical knowledge. As already observed in other studies conducted in northern Italy [5,7], the deeper integration of mountain communities within the national economy and the expansion of the health service from the end of the 1970s, along with a better access to modern drugs, could explain the erosion of TEK linked to the use of medicinal plants in the study area.

We observed during our field visits to the valley that local people are mainly dependent at present on the touristic seasons (usually during summer, and during ski period in winter), while during other periods of the year, few people permanently inhabit the villages. The development of mass tourism (leisure and sports tourism) in the Susa Valley, which also occurred in other Alpine valleys in Italy [50,51], engendered a profound transformation in the socioeconomic milieu, precipitating a transition from agrarian pursuits to engagement in the burgeoning tourist industry. This transition has been marked by a progressive detachment of the population from the local environment and, in particular, by the following human ecological changes: (1) major human migration from the upper valley to the lowlands over the past decades; (2) abandoning pastoralism (and all its derived animal products) and forest-based activities (e.g., chestnut and hazelnut collection) which were economic bases in the region; (3) the onward march of urban development, as exemplified by the expansion of touristic settlements like Bardonecchia and Sestriere, which started four decades ago. These transformations during the past few decades have significantly impacted the nexuses between local communities and their traditional activities and ultimately between the locals and their immediate landscape, and led to an erosion in the traditional ecological knowledge. Moreover, these dynamics have possibly impacted the ecological habitat of many medicinal plants, as humans’ interest in maintaining the former ecosystem, including the useful plants, has significantly decreased. For instance, the study of Lomagno and Lomagno Caramiello [17] reported four species of the genus *Prunus* which have not been reported in our study, which may be a result of the current neglect of the forest. On the other hand, the absence of some pastoralism-related species (e.g., *M. pusilla*) could also be a result of the abandonment of pastoralism-related ecosystems.

### 3.4. Economically Promising Plant Species and Their Applications in the Food and Beverage Industry, Rural Development, Gastronomic Tourism, and More

Our study results highlight several plant species that may have high economic value for their primary or secondary products. Thirteen respondents (43%) in our study reported using *Artemisia genepi* for its digestive properties. All informants agreed on the method of preparation (infusion in alcohol). Such preparation has a historical link to other *Artemisia* spp.-based preparations of the scholarly Mediterranean such as the Roman “*vinum absinthiatum*”, which can be considered the early precursor of what is known nowadays as vermouth [27]; however, as a previous study that we conducted in the upper Varaita Valley suggested [1], *genepì* was in the past mainly infused in hot milk by locals in the upper Piedmontese valleys and used as a kind of “aspirin”, and, only later, possibly with the intensification of the trade with the lowlands, became an “alcoholic” beverage (first in wine, later in spirits) [1]. Furthermore, the growing popularity of wild *Artemisia* spp.-based alcoholic preparations and especially of those that are absinth-based, which are now legal in Europe if they comply with the regulatory limits for thujones (35 mg/L) [52], further testifies to the interest of consumers in this type of beverages. The ecological availability, however, of *A. genepi* and of its related species named with the same local phytonym (*A. umbelliformis* Lam., *Artemisia lagopus* subsp. *triniana* (Besser) Korobkov) in the Alps is minimal, and they are strongly regulated by regional laws in Italy [53]. The recent domestication of *A. umbelliformis* Lam. throughout the Alps has paved the way for the ever-growing popularity of *genepì*-based products such as teas, chocolates, candies, jams, alcoholic beverages (e.g., beer infused with *A. umbelliformis* flowers), and condiments. This trend has increased the number of studies aiming to isolate compounds useful as markers to fight adulteration [27]. Altogether, Alpine wild *Artemisia* spp. are highly recommended for consideration as to their further agronomic, economic, and gastronomic potentialities. Considering that the lower Susa Valley, and especially the Piedmontese hills, are well-known for their wine production, combining locally cultivated *A. umbelliformis* and locally produced wine may have a promising economic value in the immensely growing light-alcoholic appetizing-beverage industry, i.e., the iconic Piedmontese vermouth industry. For the same purposes, and also for new possible “tonic waters”, *A. absinthium* and *Achillea* spp. could possibly also be tested and evaluated in different combinations, as well as with spices, *Citrus* spp. peels, and, especially, local wild aromatic plants that have a long history of use at the study’s site, such as *Carum carvi* fruits, *Thymus serpyllum* leaves, and aerial parts of *Tanacetum parthenium.* This latter taxon, in particular, is locally known as *arquebuse*; it is often even semi-domesticated in the local home gardens, and represents the main ingredient of a forgotten, fascinating alcoholic digestive beverage with the same name, which could be revitalized.

Other promising species in this context are the ubiquitous *T. officinale* (12 reports) and *Urtica dioica*; both plants are used as pillars of local wild gastronomy and as nutraceuticals, as well as for their medicinal interest. The leaves of *Taraxacum*, consumed raw or cooked, are perceived to be a blood purifier. *U. dioica* is widely appreciated in teas and local cuisine, cooked as spinach for its mild flavor and health benefits. The traditional use of these edible greens could be further exploited in local specialty food products and in the local restaurant/hospitality industry. Considering their agronomical and nutritional values, they could be considered for future domestication programs in the region. *Hypericum perforatum* is another species that our study participants cited frequently; the plant is prepared as tea or infused into alcohol or olive oil for external uses. Having a wide range of medicinal uses, it holds economic potential for its potential uses in herbal teas and traditional remedies. In dermatological preparation, as in many Mediterranean communities, it is used against skin burns, sunburnt skin, and skin stress, as well as for its anti-inflammatory properties. Therefore, diverse *Hypericum*-centered preparations (food supplements, herbal teas, and cosmetic products) may be promising and have an attractive economic value. Eleven respondents highlighted the species of *A. montana* for its benefits against shocks and trauma, while the oil is also used for massage against pain and rheumatism. As both *H. perforatum* and *A. montana* are prepared by infusing it into oil, this may suggest the further artisanal, small-scale use of new herbal oil plant extracts. Additionally, the small-scale production of new possible herbal teas for the gastronomic market and based on local wild plants could be the object of further feasibility explorations; this could be worthwhile for herbal teas based upon *Viola*, *Arctosphylus*, *Malva*, *Crataegus*, and *Carum* spp. aerial parts/fruits.

The two emerging trends that drive the use of herbs in the European food industry [54] are linked to certain considerations:

- Their functionality has been highlighted, as there is an increasing demand for functional food and beverages promoting gut, brain and holistic health. In our study this could open up innovative and novel uses of aromatic wild spp. (i.e., *Carum, Achillea*, and *Thymus* spp.). Moreover, in line with the increasing demand for prebiotic- and probiotic-rich foods and beverages [55], the identified species could be used in the development of lacto-fermented products based on wild plant resources (i.e., wild vegetables and seasoning spp.–based *kimchis* and *kombuchas*). Some of the above-mentioned species could also be used for the production of healthy condiments to substitute for salt and sugar. Our study possibly suggests the development of seasoning salts for the local market and the touristic one, in which the aromatic dried local wild-plant component, mixed with salt, could be used to decrease overall domestic salt intake.
- Their roles in shaping ethnic foods and beverages and in creating new sustainable opportunities for the local economy have been highlighted [56]. In the context of increasing food-tourism and the growing demand for local specialty foods and cuisine [57,58,59,60], wild plants and herbs may have potential in terms of distinguishing the local foodscape and its associated cuisine. This offers local actors the opportunity to support the continuation of food traditions and heritage through ad hoc projects in the restaurant industry and gastronomic-tourism sector, including ecotourism, with local-food-based and avant-garde restaurants focusing on the revitalization of Alpine gastronomy. Additionally, artisanal entrepreneurial ventures based on traditional wild food-plants and herbs (e.g., vegetable and fruit preserves, and condiments) and educational and community-based projects aimed at promoting the sustainable use and consumption of these resources could be also envisioned. In our study area, this opportunity could be further fostered by designing and conducting specific food-scouting projects aimed at the documentation and revitalization of local plant-based heritage foods and cuisines in neighboring areas, both within the Provencal and Franco-Provencal speaking communities.

Some sustainability issues should be considered in the future development of projects aimed at the revitalization and promotion of wild plants and herbs in our study area. It is critical to mention that some of these promising economic species have been listed on the “Red List” as being of “Least Concern”, such as *T. serpyllum* [61]. In addition, some other highly used species (e.g., *A. genepi*) have been highlighted by local people as threatened, even though they have not been listed on the Red List. Therefore, serious environmental consideration should be taken when planning to use these species on a wide economic scale, including the possibility of ad hoc local regulations to avoid over-exploitation of these natural resources.

## 4. Conclusions

Our study documented 36 species used as medicine and food-medicine in the Susa Valley of NW Italy. The study has conducted a comparison between our documented species and 96 plant species reported by a previous study conducted in the area in 1970. The comparison showed a critical decrease in the knowledge and use of medicinal plants in the region. This change was attributed to socioeconomic, urban, touristic, cultural, health system, and, possibly, environmental changes that have occurred in the region in the past half-century. However, further reasons for the documented change include differences between the methodologies applied in our study and those of the study conducted in 1970 [17]. The present study suggests several species for future applied-botany studies and projects using local plant resources in the study’s region. These economically promising species could play a crucial role in the well-being and development of the communities in NW Italy, as they could be a basis for future innovative and sustainable foods, nutraceuticals, and medicinal products. Thus, this study has expanded the scientific knowledge beneficial for increases in the competitiveness of the food system of NW Italy in terms of quality and the added value of food products.

## Figures and Tables

**Table 1:** Plant species used for medicinal purposes in the Susa Valley.

Scientific Name; Family Name	Reported in the Study in 1970 [17]	Reported in the Present Study	Local Name(s) *	Part Used *	Mode of Preparation; Medical Use *	If Species is Reported in the Present Study	
						Frequency of Citation	Medicine (M); Food-Medicine (FM)
*Achillea erba-rotta* All.; Asteraceae	Yes	No	-	The whole plant	Tea; Diuretic	-	-
*Achillea millefolium* L.; Asteraceae	No	Yes	Achillea	Flowers	Tea; Helps digestion	1	M
*Achillea nana* L.; Asteraceae	Yes	No	-	The whole plant	Tea; Diuretic	-	-
*Aconitum anthora* L.; Ranunculaceae	Yes	No	Anthòr	Roots	Cut roots; To calm the pain of intestinal colic	-	-
*Aconitum napellus* L.; Ranunculaceae	Yes	No	Erba d’el luv	Leaves and flowers	Tea; Against pain	-	-
*Aconitum anthora* L.; Ranunculaceae	Yes	No	Erba d’el luv	Leaves and flowers	Tea; Against pain	-	-
*Agrimonia eupatoria* L.; Rosaceae	Yes	No	-	The whole plant	Tea; Anti-diarrheal	-	-
*Alcea rosea* L.; Malvaceae	Yes	No	Malva rosa	Roots	Tea; Against kidney stones	-	-
*Alkekengi officinarum* Moench; Solanaceae	Yes	No	-	Fruits	Tea; Diuretic	-	-
*Allium ursinum* L.; Amaryllidaceae	No	Yes	Aglio selvatico; Aglio orsino; L’ajursino	Leaves’ juice	Raw in salad, Soup; Against arthritis	5	FM
*Allium sativum* L.; Amaryllidaceae	Yes	No	Aj	Bulbs	Crushed bulb with honey is used as a vermifuge; Tea against hypotension	-	-
*Althaea officinalis* L.; Malvaceae	Yes	No	Malva rosa	Roots	Tea; Against kidney stones	-	-
*Angelica sylvestris* L.; Apiaceae	Yes	No	Angelica	Roots	Infusion in liqueur; Digestion	-	-
*Arctium lappa* L.; Asteraceae	Yes	Yes	Bardana	Roots	Tea; Clean the blood and liver	2	M
*Arctostaphylos uva-ursi* (L.) Spreng.; Ericaceae	Yes	Yes	Uva ursina	Fruits	Raw; Good for urinary tracts	4	FM
*Arnica montana* L.; Asteraceae	Yes	Yes	Arnica; Fior di tabacco	Flowers	Infusion in oil; Against shocks and trauma, oil is also used for massage against pain and rheuma.	11	M
*Artemisia absinthium* L.; Asteraceae	Yes	Yes	Assenzio; Ford	Roots	Grinded into liqueur (infused with white wine); Anti-parasite, helps digestion, (anti-medical purpose evokes hallucinations)	4	M
*Artemisia genipi* Stechm.; Asteraceae	Yes	Yes	Genepì	Stem, Roots, and Flowers	Infusion in alcohol; Good for digestive system	13	M
*Artemisia glacialis* L.; Asteraceae	Yes	No	Genipí fumela	The whole plant	Tea; Digestion	-	-
*Artemisia umbelliformis subsp. umbelliformis;* Asteraceae	Yes	No	Genipí fumela	The whole plant	Tea; Digestion	-	-
*Brassica rapa* L.; Brassicaceae	Yes	No	Rava	Root	Sugar is left in the root for two days and then given for children suffering from whooping cough	-	-
*Calendula officinalis* L.; Asteraceae	No	Yes	Calendula	Flowers	Infusion in alcohol; Disinfectant properties	2	M
*Capsella bursa-pastoris* Medik.; Brassicaceae	Yes	No	-	The whole plant	Tea; Anti-hemorrhagic (especially for nasal hemorrhages).	-	-
*Carlina acaulis* L.; Asteraceae	Yes	No	Cardon d’ montagna	The whole plant	Tea; Used in pulmonary affections	-	-
*Carum carvi* L.; Apiaceae	No	Yes	Kumin	Seeds	Liquor; For digestion	1	FM
*Chelidonium majus* L.; Papaveraceae	Yes	Yes	Celidonia	Leaves juice	Leaves’ juice helps against warts.	1	M
*Cichorium intybus* L.; Asteraceae	Yes	Yes	Cicoria selvatica; Girasole; Cuitserce; Cicoria delle montagne	Leaves	Boiled, raw in salad; Disinfectant of urinary system	6	FM
*Crataegus monogyna* Jacq.; Rosaceae	No	Yes	Biancospino	Flowers	Tea; Against heart diseases	2	M
*Cyclamen purpurascens* Mill.; Primulaceae	Yes	No	Ciclamin	Roots	-	-	-
*Cynodon dactylon* (L.) Pers.; Poaceae	Yes	No	Cramon	The whole plant	Tea; Diuretic	-	-
*Daphne cneorum* L.; Thymelaeaceae	Yes	No	-	The whole plant	Crushed plant with oil is used as rubefacient and excitatory	-	-
*Daphne mezereum* L.; Thymelaeaceae	Yes	No	-	The whole plant	Crushed plant with oil is used as rubefacient and excitatory	-	-
*Datura stramonium* L.; Solanaceae	Yes	No	-	Leaves	Smoke of leaves against asthma attacks	-	-
*Digitalis grandiflora* Mill.; Plantaginaceae	Yes	No	-	Flowers	Tea; Diuretic	-	-
*Digitalis lutea* L.; Plantaginaceae	Yes	No	-	Flowers	Tea; Diuretic	-	-
*Dryopteris filix-mas* (L.) Schott; Dryopteridaceae	Yes	No	-	Rhizome	Chewing; Anti-bacterial	-	-
*Equisetum arvense* L.; Equisetaceae	Yes	Yes	Equiseto; Erba cavallina	Aerial part	Tea; Diuretic	1	M
*Equisetum telmateia* Ehrh.; Equisetaceae	Yes	No	Coa d’ caval	The whole plant	Tea; Diuretic	-	-
*Foeniculum vulgare* Mill.; Apiaceae	No	Yes	Finocchio selvatico	Stem, flowers, leaves	Stomachache (especially for children)	3	FM
*Fragaria vesca* L.; Rosaceae	Yes	No	Frôla	Stems	Tea; Febrifuge	-	-
*Gentiana acaulis* L.; Gentianaceae	Yes	Yes	Genzianella	Roots, flowers	Bitter liqueur; For digestion	4	M
*Gentiana lutea* L.; Gentianaceae	Yes	Yes	Genziana	Roots	Infused in alcohol or in oil; For digestion and treatment of ulcers	7	M
*Hedera helix f. helix*; Araliaceae	Yes	No	Edera	Leaves	To clean wounds; Tea against hair loss	-	-
*Hepatica nobilis Schreb.*; Ranunculaceae	Yes	No	-	Leaves	Cleaning wounds	-	-
*Hypericum perforatum* L.; Hypericaceae	Yes	Yes	Iperico; trucarań	Leaves, flowers	Tea, infused in alcohol or oil; Against skin burns, anti-inflammatory, for sunburnt skin, for skin stress, oil used for massages; As a cosmetic oil; Calming the nervous system	12	M
*Hyssopus officinalis* L.; Lamiaceae	Yes	No	Issôp	Flowers	Tea; Against cough	-	-
*Ilex aquifolium* L.; Aquifoliaceae	Yes	No	-	Flowers and fruits	Tea; Febrifuge	-	-
*Larix decidua* Mill.; Pinaceae	Yes	No	Pin	Resin	Resin mixed with oil is used as antirheumatic	-	-
*Lavandula officinalis* Chaix.; Lamiaceae	Yes	Yes	Lavanda	Whole plant	Tea for digestion; Essential oil against mosquitos’ bites	1	M
*Malva pusilla* Sm.; Malvaceae	Yes	No	Malva	Flowers and leaves	Calming, anti-neuralgic, and cough suppressant	-	-
*Malva sylvestris* L.; Malvaceae	Yes	Yes	Malva	Flowers	Tea; Anti-inflammatory; Vaginal cleanse during pregnancy; Blood purifier; Refreshing drink	5	M
*Matricaria chamomilla* L.; Asteraceae	Yes	Yes	Camomilla di montagna	Flowers	Tea; Against headache	1	M
*Mentha spicata* L. and possibly other *Mentha* spp.; Lamiaceae	Yes	Yes	Menta selvatica	Leaves	Tea; For digestion	3	FM
*Nasturtium officinale* R.Br.; Brassicaceae	No	Yes	Crescione	Aerial part	Salads; Good for blood	4	FM
*Nepeta nepetella* L.; Lamiaceae	Yes	No	-	The whole plant	Tea; Digestion	-	-
*Olea europaea* L.; Oleaceae	Yes	No	Uliva	Leaves	Tea; Hypotensive	-	-
*Papaver rhoeas* L.; Papaveraceae	Yes	No	Papaver	Leaves and petals	Tea; Calming and diaphoretic	-	-
*Parietaria officinalis* L.; Urticaceae	Yes	No	-	The whole plant	Tea; To promote peripheral circulation	-	-
*Petroselinum crispum* (Mill.) Fuss; Apiaceae	Yes	No	Pransemo	Roots	For veterinary uses	-	-
*Peucedanum oreoselinum* Moench; Apiaceae	Yes	No	-	Roots	Tea; Expectorant	-	-
*Plantago major* L.; Plantaginaceae	Yes	Yes	Plantago major	Leaves	Crushed leaves; Wounds healing	1	M
*Picea abies* (L.) H.Karst.; Pinaceae	Yes	No	Sapin	Resin	Resin mixed with oil is used as antirheumatic	-	-
*Pinguicula alpina* L.; Lentibulariaceae	Yes	No	-	Leaves	Crushed leaves with lard are used to treat bruises and sprains	-	-
*Pinguicula vulgaris* L.; Lentibulariaceae	Yes	No	-	Leaves	Crushed leaves with lard are used to treat bruises and sprains	-	-
*Pinus uncinata* Ramond ex DC.; Pinaceae	Yes	No	Pin	Resin	Resin mixed with oil is used as antirheumatic	-	-
*Pinus sylvestris* L.; Pinaceae	Yes	Yes	Pino selvatico; Pinje; Le coce	Young sprouts	Extract syrup by preserving it under sugar for 2 months; Cough treatment	2	M
*Prunus avium* (L.) L.; *Rosaceae*	Yes	No	Ceresé	Petioles	Tea; Diuretic	-	-
*Prunus domestica* L.; Rosaceae	Yes	No	Bergna	Seeds	Crushed seeds with honey are used as a vermifuge	-	-
*Prunus padus* L.; Rosaceae	Yes	No	-	Bark	Tea; Diaphoretic and febrifuge	-	-
*Prunus persica* (L.) Batsch; Rosaceae	Yes	No	Perssi	Petals	Tea; Laxative	-	-
*Pulmonaria officinalis* L.; Boraginaceae	Yes	No	-	Leaves	Tea; Calming	-	-
*Quercus robur* L.; Fagaceae	Yes	No	Rol	Acorn, bark	Tea (decoction)	-	-
*Rheum rhabarbarum* L.; Polygonaceae NW	No	Yes	Rabarbaro	Roots	Infused in white wine; For digestion	2	FM
*Rhododendron ferrugineum* L.; Ericaceae	Yes	No	-	Flowers	Tea; Kidney stones	-	-
*Rosa canina* L.; Rosaceae	Yes	Yes	Rosa canina; Gratta cü	Fruits	Marmalade, raw, tea, or infusion in liquor; Rich in vitamin C, to calm the stress; Infused in liquor, is good during sickness	3	FM
*Rubus idaeus* L.; Rosaceae	Yes	No	Ampola	Fruits	Infusion with fruits of *v. myrtillus* in wine	-	-
*Rubus ulmifolius* Schott; Rosaceae	No	Yes	More; Rove; Pun di rovi	Young leaves	Tea; For throat pain	6	FM
*Rumex alpinus* L.; Polygonaceae	Yes	No	Rebarbar d’ montagna	Leaves and flowers	Tea; Digestion	-	-
*Ruscus aculeatus* L.; Asparagaceae	Yes	No	Agrovert	Fruits	Tea; Febrifuge	-	-
*Sambucus nigra* L.; Viburnaceae	Yes	No	Sanbur	Flowers and fruits	Infusion in wine and used as cleansing drink; Flowers fried in oil and mixed with wax and fat to treat arthritis and rheumatism	-	-
*Sambucus racemosa* L.; Viburnaceae	Yes	No	Sanbur	Flowers and fruits	Infusion in wine and used as cleansing drink; Flowers fried in oil and mixed with wax and fat to treat arthritis and rheumatism	-	-
*Salix caprea* L.; Salicaceae	Yes	No	Sales	Leaves, bark, young branches	-	-	-
*Salvia* sp.; Lamiaceae	Yes	No	Salvia	Leaves	Tea; Digestion	-	-
*Sempervivum* sp.; Crassulaceae	Yes	No	-	Leaves	-	-	-
*Siler montanum* Crantz; Apiaceae	Yes	No	Apia	The whole plant	The whole plant crushed with lard is used to treat sprains	-	-
*Solanum nigrum* L.; Solanaceae	Yes	No	Erba morela	Leaves	Tea; For rheumatic pain	-	-
*Syzygium aromaticum* (L.) Merr. and L.M.Perry NW; Myrtaceae	No	Yes	Garofano	Flower buds	Tea, placing it between the teeth; Against pain in teeth and throat	1	M
*Tanacetum vulgare* L.; Asteraceae	Yes	No	T’nea	Leaves	Tea; Hypotensive	-	-
*Taraxacum officinale* F.H.Wigg.; Asteraceae	Yes	Yes	Tarassaco; Girasole; Ciguriaz	Stem, leaves, flowers	Salad, cooked; Blood purifier	12	FM
*Teucrium chamaedrys* L.; Lamiaceae	Yes	No	-	Leaves	Tea; Blood purifier	-	-
*Thymus serpyllum* L.; Lamiaceae	Yes	Yes	Timo selvatico; Timo serpillo; Timo delle montagne	Leaves, flowers	Food flavoring; Good for cough, fever, and respiratory diseases	7	FM
*Tilia cordata* Mill.; Malvaceae	Yes	No	Tilia	Flowers	Tea; Diaphoretic and cough-relieving	-	-
*Tussilago farfara* L.; Asteraceae	Yes	Yes	Tussilagine	Leaves	Tea; Against cough	1	FM
*Tragopogon pratensis* L.; Asteraceae	Yes	No	Barbaboch	Leaves	Tea; Purifier	-	-
*Urtica dioica* L.; Urticaceae	Yes	Yes	Ortica	Leaves	As food; Iron deficiency, rich in vitamins E and C	11	FM
*Vaccinium myrtillus* L.; Ericaceae	Yes	Yes	Mirtilli	Fruits	Fresh; Good for eyes	1	FM
*Valeriana locusta* L.; Caprifoliaceae	Yes	No	Sarset	Leaves	Decoction to be used warm for eye baths in conjunctivitis	-	-
*Valeriana officinalis* L.; Caprifoliaceae	No	Yes	Valeriana	Rhizome	Tea; Against anxiety	1	M
*Veratrum album* L.; Melanthiaceae	Yes	No	-	Roots	For veterinary uses	-	-
*Verbascum thapsus* L.; Scrophulariaceae	Yes	No	-	Leaves	Tea for cough; Fresh leaves are used to soothe pain of arthritis topically	-	-
*Veronica chamaedrys* L.; Plantaginaceae	Yes	No	-	Flowering pods	Tea for cough	-	-
*Veronica officinalis* L.; Plantaginaceae	Yes	No	-	Flowering pods	Tea for cough	-	-
*Veronica allionii* Vill.; Plantaginaceae	Yes	No	Te d’ montagna	Leaves	Tea; Invigorating	-	-
*Vinca minor* L.; Apocynaceae	Yes	No	-	Leaves	Tea; For gingivitis	-	-
*Viola calcarata* L.; Violaceae	Yes	No	Viola	Flowers	Tea; Anti-toxic	-	-
*Viola cenisia* L.; Violaceae	No	Yes	Viola di montagna; Viola del moncenisio	Flowers	Tea; Against cough	4	M
*Viola tricolor* L.; Violaceae	Yes	Yes	Viola del pensiero	Young aerial part	Tea; Against cold	3	M
*Viscum album* L.; Santalaceae	Yes	No	Ghi	Leaves	Tea; Hypotensive	-	-
*Zea mays* L.; Poaceae	Yes	No	Melia	-	Tea; Prostatitis	-	-

NW: Not wild; *: Data of the present study is prioritized when the taxon is reported in both studies.

**Table 2:** Aliment categories based on the respondents’ reports.

Ailment Category	The Present Study		Study Published in 1970 [17]
	Number of Reported Species	Most-Reported Species for the Relevant Category	Number of Reported Species
Digestive system disorders	10	*Artemisia genipi*	15
Respiratory system disorders	7	*Thymus serpyllum*	18
Circulatory and blood system disorders	5	*Taraxacum officinale*	11
Genitourinary system disorders	4	*Cichorium intybus*	15
Nervous system disorders	4	*Hypericum perforatum*	7
Inflammation	3	*Hypericum perforatum*	6
Pain	3	*Arnica montana*	7
Nutritional disorders	2	*Urtica dioica*	2
Musculoskeletal system disorders	2	*Arnica montana*	10
Skin/subcutaneous cellular tissue disorders	1	*Hypericum perforatum*	5

## Data Availability

The data that support the findings of this study are presented in the article.
